# Response to erlotinib in a patient with lung adenocarcinoma harbouring the *EML4-ALK* translocation: A case report

**DOI:** 10.3892/ol.2015.2897

**Published:** 2015-01-26

**Authors:** GRETA ALÌ, ANTONIO CHELLA, CRISTIANA LUPI, AGNESE PROIETTI, CRISTINA NICCOLI, LAURA BOLDRINI, FEDERICO DAVINI, ALFREDO MUSSI, GABRIELLA FONTANINI

**Affiliations:** 1 Department of Pathological Anatomy, Azienda Ospedaliera Universitaria Pisana, Pisa, Tuscany I-56126, Italy; 2 Department of Pneumology, Azienda Ospedaliera Universitaria Pisana, Pisa, Tuscany I-56126, Italy; 3 Department of Surgical, Medical, Molecular Pathology and Critical Care, Division of Pathological Anatomy, University of Pisa, Pisa, Tuscany I-56126, Italy; 4 Unit of Thoracic Surgery, Azienda Ospedaliera Universitaria Pisana, Pisa, Tuscany I-56126, Italy; 5 Department of Surgical, Medical, Molecular Pathology and Critical Care, Division of Thoracic Surgery, University of Pisa, Pisa, Tuscany I-56126, Italy

**Keywords:** *EML4*-*ALK* rearrangement, lung adenocarcinoma, erlotinib, crizotinib

## Abstract

Lung cancer is the leading cause of cancer-associated mortality worldwide, and the mainstay of treatment remains to be personalised therapy. Tyrosine kinase inhibitors of the epidermal growth factor receptor (EGFR-TKIs) have been reported to exert a significant impact in the treatment of non-small cell lung cancer (NSCLC), particularly in patients harbouring mutations in the *EGFR* gene. The echinoderm microtubule-associated protein-like 4-anaplastic lymphoma kinase (*EML4-ALK*) gene translocation has been described in a subset of patients with NSCLC and possesses potent oncogenic activity. This translocation represents one of the most novel molecular targets in the treatment of NSCLC. Patients who harbour the *EML4-ALK* rearrangement possess lung tumours that lack *EGFR* or *K-ras* mutations. The present study reports the case of a patient possessing the *EML4-ALK* rearrangement that was initially treated with erlotinib and achieved a lasting clinical response. To the best of our knowledge, the current study is the first report of a clinical response to EGFR-TKI in a patient with lung adenocarcinoma harbouring the *EML4-ALK* fusion gene, but no *EGFR* mutations. However, as the disease progressed, the *ALK* gene status of the tumour was investigated, and based upon a positive result, the patient was treated with crizotinib and achieved a complete response. In conclusion, the present study suggests that the *EML4-ALK* rearrangement is not always associated with resistance to EGFR-TKIs. Further studies are required to clarify the biological features of these tumours and to investigate the mechanisms underlying the primary resistance to EGFR-TKIs when the *EML4-ALK* rearrangement is present.

## Introduction

Despite improvements in the detection and treatment of lung cancer, it remains the most common cause of cancer-associated mortality worldwide (1). Although the mainstay of treatment for the majority of patients with advanced non-small cell lung cancer (NSCLC) continues to be cytotoxic chemotherapy (2), novel targeted therapies in NSCLC patients have been developed due to the identification of specific genetic lesions that drive the proliferation of cancer cells (3,4).

Tyrosine kinase inhibitors of the epidermal growth factor receptor (EGFR-TKIs), including gefitinib and erlotinib, have been revealed to significantly impact the treatment of patients with NSCLC. Patients harbouring mutations in the *EGFR* gene, in particular, experience a greater clinical benefit from EGFR-TKIs compared with traditional cytotoxic chemotherapy administered as first-line, second-line or maintenance therapy (5,6).

In previous studies, the echinoderm microtubule-associated protein-like 4-anaplastic lymphoma kinase (*EML4-ALK*) gene translocation has been identified in a subset of patients with NSCLC, characterized by distinct clinicopathological features, including a lower age at onset (median ago of 52 years compared with 66 years in patients with the EGFR mutation), adenocarcinoma histology and a history of never or light smoking (4–8). This fusion gene, which causes the constitutive expression of ALK, induces oncogenic cell transformation *in vitro* and *in vivo* (9). In NSCLC, the *ALK* rearrangement has been reported to be associated with distinct clinicopathological characteristics, including a lower age at onset, adenocarcinoma histology and a history of never or light smoking (8). In the majority of cases, this rearrangement and other well-known oncogenic mutations, including the *EGFR* and *K-ras* mutations, are mutually exclusive (8). Crizotinib, a small-molecule dual inhibitor of c-MET and the ALK receptor tyrosine kinase, has previously demonstrated a significant clinical benefit in patients with *EML4-ALK*-positive NSCLC in a clinical trial (10,11). Written informed consent was obtained from the patient.

## Case report

**Figure 1:**
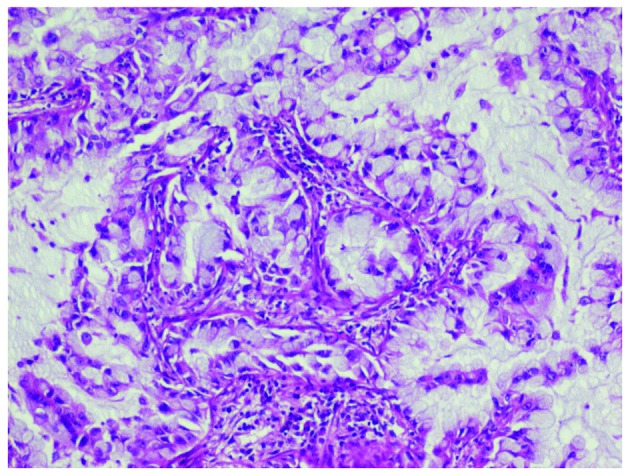
Lung adenocarcinoma exhibiting a mucinous growth pattern, areas of papillary pattern and signet ring cell features (magnification, ×10).

The present study reports the case of a never-smoking 61-year-old female that presented to the Azienda Ospedaliera Universitaria Pisana (Pisa, Italy) in June 2006 due to a persistent cough. A thoracic computed tomography (CT) revealed the presence of an irregular nodule, ~2.5 cm in diameter, in the lower lobe of the right lung. Fluorodeoxyglucose positron emission tomography (FDG-PET) examination revealed the presence of anomalous tracer uptake in the lung nodule, without pathological adenopathy. The patient underwent a right lobectomy and right superior pulmonary hilar and mediastinal lymphadenectomy. The histological diagnosis was invasive lung adenocarcinoma, with a mucinous growth pattern, areas of papillary pattern and signet ring cell features, and visceral pleura infiltration (Fig. 1). Metastases of the adenocarcinoma were observed in two carinal lymph nodes, resulting in a pathological tumour-node-metastasis (pTNM) staging of pT2aN2. Therefore, the patient underwent adjuvant radiotherapy (60 Gy) and four cycles of chemotherapy with carboplatin (6 AUC)and paclitaxel (250 mg/m^2^) for three months. The mutational status of the *EGFR* gene was analysed using single-strand conformational polymorphism (SSCP) and direct sequencing, and was found to be negative for mutations in exons 18, 19, 20 and 21 of the gene.

**Figure 2:**
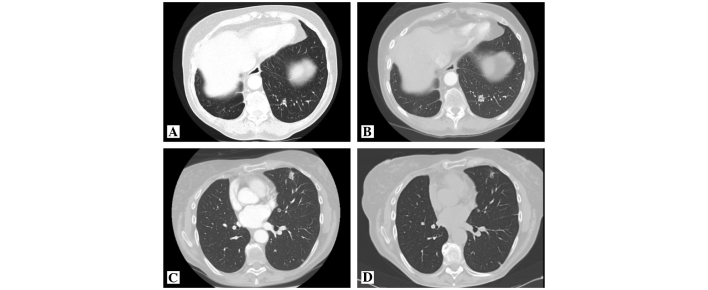
(A) Thoracic computed tomography (CT) revealing a sub-centimetre lesion, 8.0 mm in diameter, in the lower lobe of the left lung that (B) demonstrated a small increase in size after 17 months. (C) CT scan revealed progression with an increase in the size of the lesions of the left lung and (D) demonstrated a partial response following four cycles of chemotherapy with cisplatin and pemetrexed.

In January 2010, the patient exhibited disease progression (PD), with the appearance of small nodules (maximum diameter, 15.0 mm) in the upper and lower lobes of the left lung (Fig. 2A). Due to the refusal of the patient to undergo additional chemotherapy, treatment consisting of 150 mg erlotinib once a day commenced, which led to stable disease (SD) until May 2011. At that time, stereotactic radiotherapy was performed on a nodule of the lower left lobe that demonstrated a small increase in size between 15.0 and 17.5 mm (Fig. 2B). However, the patient continued treatment with erlotinib, and SD continued until June 2012 when all the lesions of the left lung demonstrated progression, with increases in size (Fig. 2C). After 28 months of treatment with erlotinib, chemotherapy with cisplatin and pemetrexed was then started. Following four cycles, a partial response (PR) was observed on a CT scan (Fig. 2D), but subsequent to six cycles, the lesions exhibited minimal progression. A second surgery was performed, and the patient underwent resection of three nodules in the left upper lobe and two nodules in the left lower lobe in December 2012. A histological diagnosis of pulmonary adenocarcinoma was made for all resected lesions.

**Figure 3:**
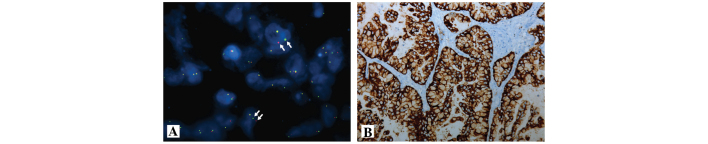
(A) Fluorescence *in situ* hybridisation analysis of the lung tumour specimen revealing cells with characteristic *ALK* translocation identified by one normal (paired) signal and two pairs of separated signals (arrows) (magnification, ×100). (B) Immunohistochemical staining for the ALK protein revealing strong granular cytoplasmic expression in the lung adenocarcinoma specimen (magnification, ×10). ALK, anaplastic lymphoma kinase.

**Figure 4:**
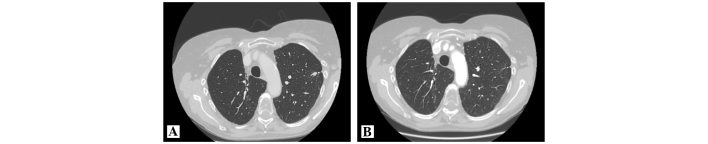
Comparison of (A) the initial computed tomography (CT) of the thorax and (B) the repeat CT three months after initiation of crizotinib therapy, demonstrating the dramatic shrinkage of the tumour lesions.

The mutational status of the *EGFR* and *K-ras* genes and the translocation status of the *EML4-ALK* fusion gene were analysed in one of the lesions of the left lower lobe. *EGFR* mutational analysis was performed using SSCP and direct sequencing, whereas pyrosequencing assays were performed for the sequence analysis of *K-ras*. The tumour did not harbour either the *EGFR* or K-*ras* mutation. Fluorescence *in situ* hybridization (FISH), which was performed using a break-apart probe for *ALK* (Abbott Molecular, Des Plaines, IL, USA), detected the presence of the *EML4-ALK* rearrangement. According to the scoring method proposed by Kwak *et al* (10), the test result for this specimen was considered positive as 38% of the neoplastic cells were positive for the *EML4-ALK* rearrangement (Fig. 3A). In addition, immunohistochemical staining of the lesion tissue using a rabbit monoclonal primary anti-ALK antibody (clone D5F3; Ventana Medical Systems, Inc., Tucson, AZ, USA) in combination with an OptiView 3,3′-diaminobenzidine Immunohistochemistry Detection kit and an OptiView Amplification kit (Ventana) revealed strong granular cytoplasmic expression of the ALK protein (Fig. 3B). Immunostaining was performed by a fully automated assay using a BenchMark XT automated slide stainer (Ventana Medical Systems, Inc.). Molecular analysis for *K-ras* mutation and the *EML4-ALK* fusion gene were also performed on the tumour resection specimen from 2006. The absence of mutation in the *K-ras* gene and the presence of the *EML4-ALK* rearrangement were confirmed by FISH and immunohistochemistry. In August 2013, a CT of the chest revealed PD with novel left lung lesions (Fig. 4A). Treatment with oral crizotinib (250 mg twice daily) was then started. A repeat chest CT (Fig. 4B) three months later demonstrated dramatic shrinkage of the lesions. The patient is continuing the treatment with crizotinib and at the date of the last follow-up (October, 2014), the patient was well and any disease recurrence was identified.

## Discussion

Treatment for lung cancer has tended towards personalised therapy. Gefitinib and erlotinib, small molecules that inhibit the tyrosine kinase activity of EGFR, are a recognized treatment for patients with advanced NSCLC. In particular, the presence of *EGFR* mutations is strongly associated with patient response to these drugs (5,6). The *EML4-ALK* fusion oncogene has previously been identified in a subset of patients with NSCLC (4–7) and represents one of the most novel molecular targets for the treatment of NSCLC. Crizotinib has resulted in an excellent clinical response in patients with *EML4-ALK*-positive NSCLC (10,11). Certain clinicopathological characteristics of *EML4-ALK*-positive NSCLCs, including a young age at onset, lack of a smoking history and adenocarcinoma histology (8), are also observed in patients with NSCLC that harbours *EGFR* mutations (3). However, the *ALK* rearrangement is thought to be mutually exclusive to other mutations in NSCLCs (7), with rare exceptions (12). In addition, the *ALK* translocation is considered to be a potential mechanism of resistance to the efficacy of EGFR-TKIs (9,12). In a preclinical study, patients with *EML4-ALK*-positive NSCLC were found to not respond to erlotinib therapy (12). Shaw *et al* (8) described the clinical response to EGFR-TKIs of 10 patients with the *EML4-ALK* rearrangement and found that none of them responded to erlotinib.

A clinical response to EGFR-TKIs in patients with *EML4-ALK*-positive lung adenocarcinoma has been previously observed in cases that harbour a coexisting *EGFR* mutation and *EML4-ALK* translocation (13–15). However, discordant results have been reported by studies investigating tumours with these concomitant molecular alterations, resulting in resistance and response to the same drug being reported by different studies (13–17).

The present study described the case of a patient with the *EML4-ALK* rearrangement that was initially treated with erlotinib and experienced a lasting clinical response. Although the ALK inhibitor is an effective drug for the treatment of *EML4-ALK*-positive NSCLC (10,11), the current patient was not initially evaluated for the presence of the translocation, as the indication was not included in clinical practice guidelines at the time of relapse (January 2010). In the absence of genetic testing for the *EML4-ALK* fusion gene, *EML4-ALK*-positive patients are likely to be treated in the same manner as patients with *EGFR* mutations. The present patient was treated with EGFR-TKI due to clinicopathological characteristics that included female gender, young age, history of non-smoking and the refusal to undergo additional chemotherapy. To the best of our knowledge, the present study is the first reported case with a clinical response to EGFR-TKIs in a patient with lung adenocarcinoma harbouring the *EML4-ALK* fusion gene and in the absence of *EGFR* mutations. At the time of the second surgery, molecular analysis was performed to investigate whether the tumour possessed the *ALK* fusion gene. The analysis was conducted on the primary and relapse tumour specimens. Notably, the *EML4-ALK* rearrangement was detected in the two specimens, but no *EGFR* mutations were identified. From the time of disease progression, the patient was treated with crizotinib and achieved a complete response, confirming that patients with this chromosomal translocation can benefit from specific ALK inhibition.

In conclusion, the present results suggest that the *EML4-ALK* rearrangement is not always associated with resistance to EGFR-TKI therapy. Future studies are required to clarify the biological features of these tumours and investigate the mechanisms underlying the primary resistance to EGFR-TKIs when the *EML4-ALK* rearrangement is present. Furthermore, the present case highlights the importance of screening patients with NSCLC by molecular testing to identify the best therapeutic approach for these patients.
